# Tools for the Recognition of Sorting Signals and the Prediction of Subcellular Localization of Proteins From Their Amino Acid Sequences

**DOI:** 10.3389/fgene.2020.607812

**Published:** 2020-11-25

**Authors:** Kenichiro Imai, Kenta Nakai

**Affiliations:** ^1^Cellular and Molecular Biotechnology Research Institute, National Institute of Advanced Industrial Science and Technology (AIST), Tokyo, Japan; ^2^The Institute of Medical Science, The University of Tokyo, Tokyo, Japan

**Keywords:** protein sorting/targeting, subcellular loalization, sorting/targeting signals, prediction methods, bacteria, archaea, eukarya

## Abstract

At the time of translation, nascent proteins are thought to be sorted into their final subcellular localization sites, based on the part of their amino acid sequences (i.e., sorting or targeting signals). Thus, it is interesting to computationally recognize these signals from the amino acid sequences of any given proteins and to predict their final subcellular localization with such information, supplemented with additional information (e.g., *k*-mer frequency). This field has a long history and many prediction tools have been released. Even in this era of proteomic atlas at the single-cell level, researchers continue to develop new algorithms, aiming at accessing the impact of disease-causing mutations/cell type-specific alternative splicing, for example. In this article, we overview the entire field and discuss its future direction.

## Introduction

Although we should not underestimate the importance of non-coding genes, the main players of the genetic system of living organisms are still regarded as protein-coding genes, which specify amino acid sequence information. Thus, in principle, we should be able to infer the *in vivo* fate of any protein from its amino acid sequence, if its environmental conditions, such as the cell type where it is synthesized, are appropriately given. For example, we should be able to predict the three-dimensional structure of a protein from its sequence or to design novel amino acid sequences that take a desired three-dimensional structure (Baker, 2019), as well as to predict how it binds/interacts with other proteins/small molecule ligands (Vakser, 2020). Another important information to be predicted is which kind of post-translational modifications, if any, it will take [at which residue(s); Audagnotto and Dal Peraro, 2017]. Also, it may be possible to predict the half-life of a given protein/peptide-based on the degradation signals (degrons) and/or other properties (Mathur et al., 2018; Eldeeb et al., 2019). Finally, the prediction of subcellular localization of a protein based on its amino acid sequence is a challenging field in bioinformatics. It is well accepted that the protein sorting for subcellular localization is regulated by so-called protein sorting (or targeting) signals, which are typically represented as a short stretch(es) of its amino acid sequence. Nowadays, many of the protein localization mechanisms/pathways that recognize and utilize such signals have been clarified. Therefore, many predictors have been developed for the recognition of such sorting signals and attempts have been done to combine such predictors, leading to the comprehensive prediction of the final localization site. However, not all such signals have been clarified. Moreover, not all proteins are equipped with such typical signals and use some alternative (minor/exceptional) pathways. Adding the knowledge of such exceptional cases will make the prediction system gradually more realistic but the objective assessment of its performance, like the ones commonly used in the field of machine learning, will become difficult because the knowledge of exceptional cases are quite unlikely to be generalized (in other words, any sequence features of such exceptional proteins, which are nothing to do with their sorting mechanisms, would work as clues for their prediction). It should be also noted that the practical value of subcellular localization predictors has been degraded because the localization information is being comprehensively determined with subcellular proteomics experiments (Harvey Millar and Taylor, 2014). However, the rise of synthetic biology as well as precision medicine will demand prediction tools that enable the prediction against artificial proteins and/or the prediction of the impact of mutations/polymorphic variations on potential sorting signals.

In this review article, we will introduce the outline of this field, emphasizing its recent progress. The readers are recommended to refer to additional reviews by other authors and ourselves, too (Imai and Nakai, 2010, 2019; Du and Xu, 2013; Nielsen, 2017; Nielsen et al., 2019).

## Prediction of Subcellular Localization Sites for Bacterial/Archaeal Proteins

Even in the simplest type of organisms, which are unicellular organisms without any subcellular compartments, proteins can be localized at either the cytoplasmic space, the cellular membrane, or the extracellular space (i.e., secreted). This is basically the case for so-called Gram-positive bacteria and archaea, but, in reality, they also have a cell wall for another localization site. The basic prediction strategy for these proteins is to combine two kinds of predictors: a predictor for N-terminal signal peptides and that for transmembrane segments. Namely, a protein that neither has an N-terminal (and cleavable) signal peptide nor any hydrophobic transmembrane segment(s) is predicted to be localized at the cytoplasmic space; a protein that has any transmembrane segment(s) (including an N-terminal uncleavable segment) is predicted to be localized at the cellular membrane; and finally, a protein that has a cleavable N-terminal signal peptide but does not have any transmembrane segment(s) is predicted to be secreted to the extracellular space or to be localized at the cell wall. In Gram-positive bacteria, proteins that are anchored to the cell wall are characterized with the existence of the LPXTG-motif, followed by a hydrophobic domain and a tail of positively-charged residues (for recent review, see Siegel et al., 2017). On the other hand, Gram-negative bacteria contain one more membrane, the outer membrane, instead of the cell wall. Therefore, their possible localization sites are the cytoplasmic space, the inner membrane (which is equivalent to the membrane of Gram-positive bacteria), the periplasm, the outer membrane, and the extracellular space. Generally speaking, proteins that are localized at the latter three sites (the periplasm, the outer membrane, and the extracellular space) have an N-terminal cleavable signal peptide but do not have any hydrophobic transmembrane segment(s). Proteins that are integrated into the outer membrane are typically β-barrel proteins (Bakelar et al., 2017). To distinguish these three types of proteins, their difference in amino acid composition and/or *k*-mer frequency as well as motif/homology-based methods are often used.

A pioneering work to propose the above formalism is published in 1991 (Nakai and Kanehisa, 1991), where the predictor was named PSORT (I). In 2003, its approach was inherited and elaborated by Fiona Brinkman’s group (Gardy et al., 2003); their software is named PSORTb (or PSORT-B). Its latest version is PSORTb 3.0 (Yu et al., 2010). The group published an excellent review of bacterial protein subcellular localization in 2006 (Gardy and Brinkman, 2006). According to the assessment shown in the review, PSORTb was the best predictor at that time. The group also releases PSORTdb, which contains a collection of experimentally-determined information of subcellular localization as well as systematic outputs of PSORTb applied to thousands of bacterial proteomes [its latest reference reports v. 3.0: (Peabody et al., 2016) but its latest version is v. 4.0]. The same group also proposes PSORTm, a variant of PSORTb designed for the prediction of metagenomic data (Peabody et al., 2020). The basic idea of PSORTm is to first identify the taxonomy of each read based on a reference database of microbial proteins. From the estimated taxonomy, the read is automatically classified with cell envelope types and then it is subject to a variant of PSORTb, which uses various types of analyses (such as motif/profile analysis) for its subcellular localization prediction. Although the assessment of its precise accuracy would be difficult, they report an assessment using artificial data and the comparison with the prediction against pre-assembled data. In view of the rapid growth of microbiome analyses, the need of characterizing metagenome data should increase even more and thus the field looks promising. Of course, other groups have developed a variety of predictors for bacterial/archaeal proteins, among which PSO-LocBact (Lertampaiporn et al., 2019), GPos-ECC-mPLoc/Gneg-ECC-mPLoc (Wang et al., 2015), BUSCA (Savojardo et al., 2018b), which will be introduced below, and ClubSub-P (Paramasivam and Linke, 2011) are released relatively recently. Some of them claim that they can deal with proteins with multiple-locations. Although once a database for (eukaryotic) proteins with multiple subcellular localizations is released (Zhang et al., 2008), it still seems difficult to classify multiple localizations objectively and quantitatively because the data come from different sources which rely on different experimental conditions (but see the discussion below).

Beyond the basic scheme described above, there are several issues to be further explored. One is the prediction of several specialized localization sites, such as host-associated, type III secretion, fimbrial, flagellar, and spore. In PSORTb, they are treated as subcategories. Of course, it is favorable that a predictor can deal with such localization sites but it is questionable if such a predictor can also deal with artificial proteins that are transported to such locations. In other words, it is likely that such predictions are easily done with simple homology transfer from known examples. Another issue is how to deal with the proteins that are transported with minor pathways. For the users’ convenience, it is desirable that a predictor can inform users which pathway the input protein will use. For example, it is surely useful if a predictor informs us that the input protein will be transported *via* the twin-arginine translocation pathway (Palmer and Stansfeld, 2020) or the lipoprotein signal peptidase II-dependent pathway (El Arnaout and Soulimane, 2019). This can already be done with several predictors, including SignalP-5.0 (Almagro Armenteros et al., 2019, see below). Hopefully, more knowledge of various protein sorting pathways should be incorporated into predictors, even if the objective assessment of their predictability would become difficult. In this sense, more benchmarking efforts/systematic analysis of subcellular localization from various viewpoints would be valuable (Stekhoven et al., 2014; Orioli and Vihinen, 2019; see below).

## Prediction of Subcellular Localization Sites for Eukaryotic Proteins

**Figure 1:**
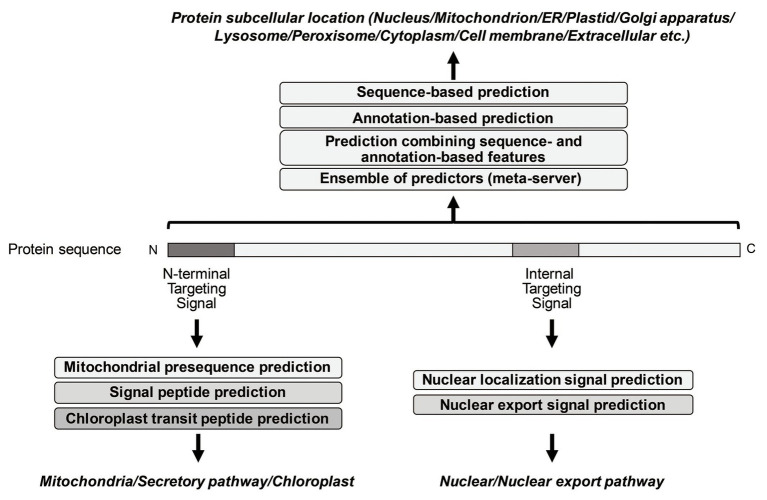
Summary of representative prediction approaches of different subcellular localization.

So far, many prediction methods of eukaryotic protein subcellular localization have been developed. They are mainly based on biological/empirical sequence features related to subcellular localization. In these methods, a variety of machine learning algorithms, such as the *k*-nearest neighbor (*k*-NN) classifier, the Random Forest classifier, the support vector machine (SVM), and the deep learning, have been used. Those methods usually target 10 main localization sites, where subcompartments of localization sites are merged into 10 major sites in order to increase the number of proteins per localization site (see Table 1). As further explained below, for the prediction of subcellular localization sites, three types of prediction features are generally used: targeting signal features, sequence-based features, and annotation-based features (Figure 1). The features associated with targeting signals are most powerful, when available, and many subcellular localization predictors based on targeting signal features have been developed. Thus, we first overview the representative targeting-signal predictors and then predictors for localization sites.

**Table 1:** Representative subcellular locations covered by predictors for eukaryotic proteins.

Main location	Representative subcompartments
Nucleus	inner and outer membranes, matrix, chromosome, nucleus speckle, etc.
Mitochondrion	inner and outer membranes, matrix, intermembrane space
Endoplasmic reticulum (ER)	ER membrane and lumen, microsome, rough ER, smooth ER, etc.
Plastid	inner and outer membranes, stroma, thylakoid, etc.
Golgi apparatus	Golgi apparatus membrane, lumen
Lysosome/Vacuole	vacuole lumen and membrane, lysosome lumen and membrane, etc.
Peroxisome	matrix, membrane
Cytoplasm	cytosol, cytoskeleton
Cell membrane	cell membrane, cell projection, apical, basal, etc.
Extracellular	–

### Prediction of Targeting Signals

The targeting signals are roughly grouped into two categories: N-terminal targeting signals and non-N-terminal targeting signals. The mitochondrial targeting signal (presequences), the signal sequence for the secretory pathway (signal peptides), and the transit signal for chloroplast (transit peptides) are well-known as N-terminal targeting signals, while the nuclear localization signal (NLS) and the nuclear export signal (NES) are internal signal sequences. Peroxisome matrix proteins contain peroxisomal targeting signal type 1 (PTS1) in the C-terminus.

#### Prediction of Mitochondrial Targeting Signal

Mitochondria have been estimated to host 1,000 to 1,500 distinct proteins. Approximately, 99% of mitochondrial proteins are encoded in the nuclear genome and are imported by translocases in the mitochondrial outer and inner membranes. Approximately 60% of mitochondrial proteins possess an N-terminal cleavable targeting signal (presequence; Vögtle et al., 2009). These presequences are typically recognized by the translocase of the outer membrane (TOM) receptors, which consist of Tom20 and Tom22, in the TOM complex. Then, they direct the translocation of signal-containing proteins through the main protein translocation channel, Tom40 (Pfanner et al., 2019). Upon translocation across the outer membrane, the presequence-containing proteins are transferred across the inner membrane by the translocase of the inner membrane complex (TIM23) with the presequence translocase-associated motor (PAM). The length of presequences is 20–60 amino acid residues (Calvo et al., 2017). The representative features of presequences are high and low composition of arginine residues and negatively-charged residues, respectively (von Heijne, 1986; Schneider et al., 1998). Positively charged amphiphilicity (amphiphilic α-helical structure with hydrophobic residues on one face and positively-charged residues on the opposite face) is also a well-characterized feature (Chacinska et al., 2009; Fukasawa et al., 2015). Recently, the TOM complex structure was revealed by cryo-electron microscopy and it provided structural insights into the import path of precursor protein containing presequence through the TOM complex (Araiso et al., 2019). Presequence is typically cleaved by three mitochondrial peptidases in the matrix (MPP, Icp55, and Oct1; Mossmann et al., 2012). The cleavage by MPP occurs after the position of two amino acids of C-terminal to an arginine (the R-2 motif). Icp55 and Oct1 subsequently cleave off one amino acid and eight amino acids from the newly-emerged N-terminus, respectively. Therefore, proteins processed by MPP and Icp55 have an arginine at position -3 (the R-3 motif) in the presequence, while proteins processed by MPP and Oct1 have an arginine at position -10 (the R-10 motif).

MitoProtII (Claros, 1995), TargetP (Emanuelsson et al., 2000), Predotar (Small et al., 2004), TPpred3.0 (Savojardo et al., 2015), and MitoFates (Fukasawa et al., 2015) were widely used presequence prediction methods. Those are developed using machine-learning techniques with these features of presequences. Those tools are also capable of predicting the existence of presequence as well as their cleavage site. MitoProtII and MitoFates are specific predictors for (mitochondrial) presequences, while TargetP, Predotar, and TPpred3.0 can also predict other N-terminal targeting signals, such as secretory signal sequence and chloroplastic targeting signal. Recently, TargetP2.0 is developed as a deep learning model, using bidirectional long-short-term memory (LSTM) and a multi-attention mechanism (Armenteros et al., 2019). Among existing tools, three of them (MitoFates, TPpred3.0, and TargetP2.0) perform better in the prediction of both the presequence existence and its cleavage site. MitoFates employs an SVM classifier by combining amino acid composition and physicochemical properties with positively charged amphiphilicity, discovered presequence motifs, and position-weight matrices of cleavage site patterns. TPpred3.0 is a combination of a Grammatical Restrained Hidden Conditional Random Field, N-to-1 Extreme Learning Machines, and SVMs. We compared the performance of those three methods, using recent proteomic data of the N-termini of mouse mitochondrial proteins (we omitted proteins whose length of cleaved N-terminal sequences is shorter than 10 or longer than 100 amino acids in the comparison; Calvo et al., 2017). The recalls of presequence prediction by TPpred3.0, MitoFates, and TargetP2.0 are 63.2, 75.9, and 79.9%, respectively. Whereas the recalls of the cleavage prediction by TPpred3.0, MitoFates, and TargetP2.0 are 27.0, 28.8, and 45.5%, respectively. MitoFates and TargetP2.0 show better performance on the presequence prediction. In the cleavage site prediction, TargetP2.0 far outperformed other methods, though the cleavage site prediction is still a challenging task. About 20% of mouse cleavage site data does not match with the R-2, R-3, and R-10 motifs (Calvo et al., 2017). It will be necessary to better characterize these untypical presequences.

#### Prediction of Signal Sequence

The targeting signal sequence for the secretory pathway (signal peptides) is located at the N-terminal of protein sequence in both eukaryotes and prokaryotes. The length of signal peptides is 16–30 amino acid residues. It is estimated that about 10–20% of eukaryotic proteome and 10% of bacterial proteome have the signal peptide at N-terminus (Kanapin et al., 2003; Ivankov et al., 2013). In eukaryotic cells, the signal recognition particle (SRP) co-translationally recognizes signal peptides upon their emergence from the ribosome and transfers them to the Sec61 translocon in the endoplasmic reticulum (ER) membrane *via* the SRP receptor (Nilsson et al., 2015). The signal peptidase cleaves off signal peptides and thus mature proteins are generated. Signal peptides share several characteristic features (von Heijne, 1990); they have tripartite architecture: a positively charged N-terminus (n-region), a hydrophobic segment (h-region), and a cleavage site for signal peptidase (c-region). The cleavage site is characterized by the (-1, -3) rule; amino acids with small, uncharged side chains at the -1 and -3 position relative to the cleavage site.

For predicting signal peptides and their cleavage sites, many prediction methods, such as SignalP 4.0 (Petersen et al., 2011), SPEPlip (Fariselli et al., 2003), Phobius (Krogh et al., 2007), and DeepSig (Savojardo et al., 2018a), have been developed. The discrimination between secretory and non-secretory proteins based on the signal peptide prediction has been most successful in targeting signal predictions because SignalP 3.0 has already achieved the best Matthews’ Correlation Coefficient (MCC) of 0.76 in eukaryotic data sets in an assessment study in 2009 (Choo et al., 2009). Recently, SignalP has been further improved as a deep neural network-based method, combining with conditional random field classification and optimized transfer learning (SignalP-5.0; Almagro Armenteros et al., 2019). According to their benchmark results, SignalP-5.0 outperforms other methods in predicting both the signal peptide existence and the cleavage site: the MCC was 0.88 in the signal peptide prediction and the recall of cleavage site detection was 72.9%.

#### Prediction of Chloroplastic Targeting Signal

The translocons at the outer and the inner membranes of chloroplasts, the TOC and TIC complexes mediate the targeting and import of ~3,500 different nuclear-encoded proteins. Those proteins are imported from the cytoplasm *via* interaction between their cleavable, N-terminal chloroplast targeting signal (transit peptides), and the TOC–TIC import systems (Li and Chiu, 2010; Paila et al., 2015). The transit peptide is removed off by the activity of stroma processing peptidase (SPP), which is related to the mitochondrial peptidase, MPP. SPP does not interact stably with the TOC–TIC import system, thus the cleavage event occurs after protein translocation or upon the emergence of the transit peptide cleavage site into the stroma. Chloroplast transit peptides are mostly unstructured but can form α-helical structures in hydrophobic environments (Bruce, 2001; Jarvis, 2008). In addition, chloroplast transit peptides have a high content of hydroxylated amino acids (e.g., serine residues) and positively charged amino acids and a very low content of negatively charged amino acids (Bhushan et al., 2006). Transit peptides and presequences are therefore similar in several aspects. In spite of the similarities, chloroplast transit peptides direct precursor proteins specifically to chloroplasts. Ge et al. (2014) demonstrated that transit peptides and presequences can be discriminated by their charge properties and hydrophobicity. Also, the analysis of 916 chloroplast proteins revealed an N-terminal domain beginning with Met-Ala and the low composition of arginine in the N-terminal portion (Zybailov et al., 2008). Moreover, Lee et al. (2019) recently showed that mitochondrial or chloroplast targeting specificities are characterized by the N-terminal regions of these targeting signals: an N-terminal multiple-arginine motif was identified as the mitochondrial specificity factor and chloroplast evasion signal. Cleavage sites of transit peptides are characterized by higher content of Ala, Ile, Cys, and Val residues (Gavel and von Heijne, 1990). The three motifs, [V,I][R,A]↓[A,C]AAE, S[V,I][R,S,V]↓[C,A]A, and [A,V]N↓A[A,M]AG[E,D], are derived by a set of 198 cleavage sites (Savojardo et al., 2015).

The existing prediction tools for the chloroplastic targeting signal deal with cleavable N-terminal transit peptides. Widely used prediction methods have been integrated as a part of prediction of N-terminal targeting signals in general: e.g., TargetP (Emanuelsson et al., 2000), iPSORT (Bannai et al., 2002), Predotar (Small et al., 2004), and TPpred3 (Savojardo et al., 2015). Among those tools, TPpred3 achieved better performance for transit peptide prediction (46% precision and 64% recall). As mentioned above, TargetP is recently updated to version 2.0 as a deep learning model (TargetP2.0; Armenteros et al., 2019). In their comparison, the precision and recall of chloroplastic transit peptide identification of TargetP2.0 are 90 and 86%, respectively, while those of TPpred3 are 76 and 69%. In the cleavage site prediction, the recalls of TargetP2.0 and TPpred3 are 49 and 30%, respectively. Like mitochondrial presequence prediction, the cleavage site prediction of chloroplastic targeting signal is a difficult problem. Comparing with the data size of signal peptides, that of transit peptides is quite small and thus the lower performance could have been caused by this reason. Larger-scale N-terminal proteomics data of chloroplastic proteins would be necessary for the improvement of their cleavage site prediction.

#### Prediction of Nuclear Localization Signals and Nuclear Export Signals

Nuclear proteins are transported into or out of the nuclei through the nuclear pore complex by the importin-β (Impβ) family nucleocytoplasmic transport receptors (Kimura and Imamoto, 2014). The human proteome contains 20 Impβ family proteins: 10 are nuclear import receptors (importin-β, transportin-1, -2, -SR, importin-4, -5 (RanBP5), -7, -8, -9 and -11), seven are export receptors (exportin-1 (CRM1), -2(CAS/CSE1L), -5, -6, -7, -t, and RanBP17), two are bi-directional receptors (imporin-13 and exportin-4), while the function of remaining RanBP6 is undetermined (Kimura and Imamoto, 2014). Those nucleocytoplasmic transport receptors are thought to recognize specific targeting signals on those cargo proteins. Several types of NLSs and NESs have been reported, so far. The most studied NLS is the classical NLS (cNLS) that binds to Impα, which is a cargo-binding adaptor exclusively used for Impβ (Lange et al., 2007). Sequences similar to the Impβ binding (IBB)-domain in Impα act as NLSs that bind directly to Impβ. Other known NLSs/NESs that bind directly to Impβ family are: the PY-NLS for Trn1 and Trn2 (Lee et al., 2006), the Leu-rich NES for CRM1 (Hutten and Kehlenbach, 2007), the SR-domain for TrnSR (Maertens et al., 2014), and the β-like importin binding (BIB)-domain, which binds to several nucleocytoplasmic transport receptors (Jäkel and Görlich, 1998). In addition, the RG/RGG-rich segment for Trn1 and the RSY-rich segment for TrnSR were reported recently (Bourgeois et al., 2020). However, these known NLSs/NESs do not explain all of the cargo recognition sites. Moreover, recent proteomic analysis for the identification of cargo proteins of 12 nucleocytoplasmic transport receptors (10 nuclear import receptors and 2 bi-directional receptors; Kimura et al., 2017) also pointed out that about 30% of identified cargos are shared by multiple receptors. The degree of multiplicity and diversity of cargo recognition by nucleocytoplasmic transport receptors are still controversial.

Among known nuclear targeting signals, cNLS and NES of CRM1 are well characterized. Thus, existing prediction methods of NLSs and NESs mainly target these two types. cNLSs are grouped into monopartite and bipartite NLSs. Monopartite NLS is characterized with a single stretch of basic residues (e.g., KR[K/R]R and K[K/R]RK), while bipartite NLS has two clusters of basic residues, separated by a spacer region of 10–12 amino acids (e.g., KRX_10–12_K[K/R][K/R]; Kosugi et al., 2009). Lisitsyna et al. (2017) assessed the prediction performance of widely used methods, Nucpred (Brameier et al., 2007), cNLSmapper (Kosugi et al., 2008a), NLStradamus (Ba et al., 2009), NucImport (Mehdi et al., 2011), and SeqNLS (Lin and Hu, 2013), using a human NLS dataset (Lisitsyna et al., 2017). NucPred, seqNLS, and NLStradamus showed better MCCs (~0.3); however, the recalls of those methods were still ~45%. Recently, Guo et al. (2020) reported INSP, which is a NLS predictor based on a multivariate regression model integrating PSSM-based conservation score, protein language-based SVM learning score, disorder-based structural score, and amino acid physical chemistry property-based score. On their test dataset, INSP showed 50.6% precision at 67.0% recall, whereas seqNLS, NLStradamus, and cNLSmapper obtained 60.6% precision at 36.4% recall, 53.9% precision at 35.6% recall, and 50.9% precision at 50.9% recall, respectively. INSP showed a favorable balance between the prediction precision and recall, but NLS prediction seems to be still difficult because the cNLS sequence patterns are often observed in non-nuclear protein sequences (i.e., false positives).

Nuclear export signals function as essential regulators for the export of hundreds of distinct cargo proteins by interacting with CRM1. So far, 11 consensus patterns of NES have been proposed by a peptide-library study and structure analyses of CRM1-NES (Kosugi et al., 2008b; Fung et al., 2015, 2017). In general, NESs are represented by *Φ*0-*x*_1-2_-*Φ*1-(*x*)_2-3_-*Φ*2-(*x*)_2-3_-*Φ*3-*x*-*Φ*4 (*Φ*1-4 denote Leu, Val, Ile, Phe, and Met while x is any amino acid. *Φ*0 is not restricted to the hydrophobic amino acids). Those hydrophobic residues in *Φ*0–*Φ*4 are bound to the corresponding hydrophobic pockets in CRM1. Based on the pattern of these *Φ*’s and spacing sequences, the NES motifs are classified into seven classes and four additional reverse classes, representing binding in the opposite direction. Several prediction tools for NESs, such as NetNES (La Cour et al., 2004), NESsential (Fu et al., 2011), NESmapper (Kosugi et al., 2014), Wregex (Prieto et al., 2014), LocNES (Xu et al., 2015), and NoLogo (Liku et al., 2018) have been developed, representing the consensus sequences with regular expressions or PSSMs as well as biophysical properties (disorder propensity, solvent accessibility, and secondary structure information). Among those tools, LocNES outperformed other prediction tools; however, the precision is ~50% at 20% recall. The low performance is caused by high false-positive rates. As mentioned above, the NES consensus patterns are simple and commonly observed in other protein sequences. Thus, it seems to be difficult to improve the prediction performance by only using the sequence information. Recently, Lee et al. (2019) provided a comprehensive table for cargo proteins, containing the location of the NES motifs with the disordered propensity, the predicted secondary structures, and the conserved domain information. They also proposed a structure modeling-based prediction which predicts the binding energy of the NES peptide bound to the binding groove of CRM1, using multiple structures of CRM1-NES peptide complex as templates (Lee et al., 2019). The structure-based methods performed at the same level as LocNES in recall rate but outperformed LocNES in specificity and false-positive rate. Thus, combining sequence-based and structure-based predictions seems promising in significantly improving the NES prediction. Moreover, NLSdb, which is a database containing NLSs and NESs, has been recently updated (Bernhofer et al., 2018). In this update, the potential set of novel NLSs and NESs has been generated by an *in silico* mutagenesis protocol. Then, the potential NLSs and NESs match at least one nuclear protein but do not match any non-nuclear proteins. The updated NLSdb contains 2,253 NLSs (1,614 are potential NLSs) and 398 NESs (192 are potential NESs). The data would be useful to further improve the NLS and NES prediction performances.

### Prediction of Subcellular Localization Site of Protein in a Cell

Existing methods for predicting subcellular localization sites can be grouped into four categories. The first category of prediction methods uses only sequence-based features. Some sequence-based features are used in localization site prediction because their differences are empirically known to be correlated with the differences between localization sites. Such empirical features include the frequency of dipeptides, *n*-grams, and *k*-mers as well as the pseudo amino acid composition of the entire amino acid sequence (or that of predicted mature sequence). Pseudo amino acid composition is more informative in terms of incorporating sequence-order information of a protein sequence (Chou, 2001). These empirical sequence-based features have also been popular in various amino acid sequence-based predictions. Besides these systematically defined features, sequence features of various known targeting signals are more or less useful, as mentioned above. Functional motifs are also used in the prediction because sequence motifs associated with the function of a protein are closely related to its localization site (for example, a protein containing a DNA-binding motif is likely to be localized in the nucleus). The first sequence-based method was PSORT (I) (Nakai and Kanehisa, 1992), which was developed about 30 years ago, and later many other methods, such as WoLF PSORT (Horton et al., 2007), CELLO2.5 (Yu et al., 2006), and DeepLoc (Almagro Armenteros et al., 2017), have been developed. WoLF PSORT is an update of PSORT II (Horton and Nakai, 1997), which converts the input amino acid sequences into a numerical vector consisting of amino acid composition and PSORT/iPSORT (Nakai and Kanehisa, 1992; Bannai et al., 2002) localization features, and then classifies proteins into subcellular locations with a weighted *k*-NN classifier. CELLO2.5 is a two-level SVM classifier system: the first level comprises a number of SVM classifiers, each based on distinctive sets of feature vectors generated from amino acid sequence data, and the second level SVM classifier functions as the jury machine to generate the probability distribution of decisions for possible localizations. Recently, several deep learning-based predictors are developed. DeepLoc is their representative. DeepLoc uses recurrent neural networks (RNNs) with long short-term memory (LSTM) cells that process the entire amino acid sequence and an attention mechanism identifying sequence regions important for the subcellular localization.

The second category of predictors uses annotation-based features obtained from experimental evidence. GO terms, localization annotation in UniProt, functional domain, protein-protein interaction, and literature information from PubMed abstracts are categorized into this type of features. mGOASVM (Wan et al., 2012) is a predictor for the subcellular localization of multi-location proteins based on GO-terms. In mGOASVM, multi-label GO vectors, which are the occurrences of GO terms of homologous proteins, are constructed, and then GO vectors are recognized by SVM classifiers equipped with a decision strategy that can produce multiple-class labels for a query protein. pLoc-mEuk (Cheng et al., 2018) is recently developed by extracting the key GO information into “Chou’s general Pseudo Amino Acid Composition.” pLoc-mEuk can also deal with proteins with multiple locations. Generally speaking, however, compared with those features, the transfer of localization annotation from homologous protein seems to be simpler and more useful. We previously pointed out that a simple homology-based inference outperforms methods based on machine learning if a homologous protein with localization annotation is available (Imai and Nakai, 2010).

The third category is the predictors combining sequence-based and annotation-based features, such as MultiLoc2 (Blum et al., 2009), SherLoc2 (Briesemeister et al., 2009), YLoc (Briesemeister et al., 2010), and LocTree3 (Goldberg et al., 2014). MultiLoc2 utilizes an SVM predictor, MultiLoc (Höglund et al., 2006), which is based on overall amino acids and the presence of known sorting signals, combined with phylogenetic profiles and GO terms. SherLoc2 combines MultiLoc2 and EpiLoc (Brady and Shatkay, 2008), a prediction system based on features derived from PubMed abstracts. YLoc is based on a simple naive Bayes classifier, which combines various features ranging from simple amino acid composition to annotation information, like PROSITE domains, and GO terms from close homologs. LocTree3 improves over a machine learning-based predictor, LocTree2 (Goldberg et al., 2012), by the combination of the machine learning-based method with a homology-based inference transfer through PSI-BLAST.

The fourth category is the ensemble of several prediction methods (meta-servers), which collects prediction scores of several predictors, and then they are trained by a machine learning technique, such as the Random Forest classifier and SVM. SubCons (Salvatore et al., 2017) is a recent ensemble method, which combines four predictors (CELLO2.5, LocTree2, MultiLoc2, and SherLoc2) using a Random Forest classifier. BUSCA also integrates different prediction methods. Prediction pipeline of BUSCA consists of predictors for targeting signals [DeepSig (Savojardo et al., 2018a) and TPpred3 (Savojardo et al., 2015)], for GPI-anchors [PredGPI (Pierleoni et al., 2008)], for transmembrane domains [ENSEMBLE3.0 (Martelli et al., 2003) and BetAware (Savojardo et al., 2013)], and for discriminators of subcellular localization of both globular and membrane proteins [BaCelLo (Pierleoni et al., 2006), MemLoci (Pierleoni et al., 2011), and SChloro (Savojardo et al., 2017)].

### Recent Benchmarks for Subcellular Localization Prediction

Evaluation of prediction performance of subcellular localization prediction is often difficult due to the following reasons: (i) There are often overlaps between their own training data and the test data of different methods. In those cases, the performances could be overestimated. (ii) Comparison of sequence-based methods with annotation-based methods or methods combining sequence- and annotation-based methods tends to be unfair. For example, the measured accuracy of annotation-based methods would become apparently higher if the majority of test data used for sequence-based methods are included in the databases used for the prediction by annotation-based methods.

To evaluate the prediction performance with less bias, Salvatore et al. recently made a benchmark dataset which consists of proteins containing identical subcellular annotations in at least two out of the three resources (Salvatore et al., 2017): two large-scale study data on subcellular localization of human proteins (Uhlen et al., 2010; Fagerberg et al., 2011; Breckels et al., 2013; Christoforou et al., 2014) and proteins with “manually curated” annotation of subcellular localization in UniProt (UniProt Consortium, 2019). Then, they examined the performance of six state-of-the-art methods [CELLO2.5 (Yu et al., 2006), LocTree2 (Goldberg et al., 2012), MultiLoc2 (Blum et al., 2009), SherLoc2 (Briesemeister et al., 2009), WoLF PSORT (Horton et al., 2007), and YLoc (Briesemeister et al., 2010)] as well as SubCons (Salvatore et al., 2017) for eight localization sites (nucleus, mitochondria, ER, Golgi apparatus, lysosome, peroxisome, plasma membrane, and cytoplasm). They used the Generalized Squared Correlation (*GC*^2^; Baldi et al., 2000) for performance evaluation. *GC*^2^ is a subtype of Gorodkin measure (Gorodkin, 2004), which can be seen as a generalization of MCC that applies to *K*-categories. The Gorodkin measure is more informative than the accuracy measure when there is an imbalance of classes. For *K* = 2, the Gorodkin measure squared is *GC*^2^. In this assessment, SubCons showed the best overall prediction performance, *GC*^2^ = 0.32, and the second best was SherLoc2 (*GC*^2^ = 0.27). On the other hand, during the development of DeepLoc (Almagro Armenteros et al., 2017), the authors made an independent test set by performing a stringent homology partitioning against experimentally annotated protein data in UniProt. Homologous proteins that fulfill a certain threshold of similarity were clustered, and then each cluster of homologous proteins was assigned to one of the five folds, ensuring that similar proteins were not mixed between the different folds. Four were used for the training and validation while the remaining one for testing. Using the test set, they compared the prediction performance of DeepLoc with the above six methods (CELLO2.5, LocTree2, MultiLoc2, SherLoc2, WoLF PSORT, and YLoc) and iLoc-Euk (Chou et al., 2011) in 10 localization sites (extracellular and plastid are added into the above eight localization sites). DeepLoc showed the best Gorodkin measure of 0.735, and the second and third best were achieved by iLoc-Euk at 0.682 and YLoc at 0.533, respectively.

Although efforts to evaluate the prediction performance with less bias have been made, more efforts seem to be necessary. According to recent benchmarking reports based on human data sets and membrane proteins (Orioli and Vihinen, 2019; Shen et al., 2020), sequence-based methods tend to show lower performance than annotation-based methods, including meta methods. However, a certain number of proteins (or their highly homologous ones) in the benchmark test data seem to be included in the database used in annotation-based methods. In addition, methods trained and tested with newly constructed data tend to show better performance because older data tend to include more mislabeled or questionable examples. Indeed, Almagro Armenteros et al. (2017) pointed out a considerable decrease of experimentally confirmed proteins in UniProt after a major change in the annotation standards on release 2014_09. The prediction performances of machine learning algorithms significantly depend on the datasets used. Some of the previously developed methods may outperform newer methods when they are trained and tested with the latest datasets. For fair assessments, performance comparison should therefore be done in each category with standardized benchmark data sets, ensuring independence between training and test data sets. Unfortunately, to the best of our knowledge, such standardized benchmark data sets have not been constructed so far. The data sets used in previous studies are often used in the development of novel methods. The standardization of prediction performance comparison is a big challenge but this is essential and important in this field. Recent progress in proteome-wide subcellular protein mapping (see below) would provide substantial information on the subcellular localization of unverified or unseen proteins as well as the information for correcting mislabeled proteins, which should be helpful in constructing standardized benchmark data sets, obviously.

## Protein Localization Resources Obtained From Recent Spatial Proteomics Approaches

Proteomics data for capturing the spatial distribution of proteins at the subcellular level (subcellular protein mapping) are useful resources for their predictive studies. Recent advances in high-throughput microscopy, quantitative mass spectrometry (MS), interactome mapping, and machine learning applications for data analysis have enabled proteome-wide subcellular protein mapping (Lundberg and Borner, 2019; Borner, 2020). Three experimental approaches are generally used for spatial proteomics: proteome-wide imaging of protein localization, protein–protein interaction network analysis, and MS-based organelle profiling. All of these approaches have produced numerous available data of human protein subcellular localization. The Human Cell Atlas provides an invaluable resource of imaging data at a single-cell level (localization of 12,003 proteins; Thul et al., 2017). The global organellar map based on biotin identification (BioID) data is now available as a resource of protein–protein interaction network analysis (4,145 proteins; Go et al., 2019). Several organelle profiling resources are obtained from fibroblasts (2,533 proteins; Jean Beltran et al., 2016) and cell lines: HeLa (8,710 proteins; Itzhak et al., 2016), five different cancer cell lines (12,418 proteins; Orre et al., 2019), and U-2 OS (2,412 proteins; Geladaki et al., 2019). In addition, organelle profiling resources of mouse primary neurons (Itzhak et al., 2017), mouse liver (Krahmer et al., 2018), mouse pluripotent stem cell (Christoforou et al., 2016), rat liver (Jadot et al., 2017), and *Saccharomyces cerevisiae* (Nightingale et al., 2019) are also available.

Each of these approaches has its own merits for the identification of protein localization: the imaging approach provides multiple localizations and has a single-cell resolution while the MS-based approach can provide peptide-level resolution and reveal the differential localization of splicing isoforms, proteolytically processed forms, and the isoforms *via* differential post-translational modifications. A recent imaging-based large-scale study reports that about a half of all proteins are localized at multiple compartments, suggesting that there is a shared pool of proteins even among functionally unrelated organelles (Thul et al., 2017). Prediction of proteins that exist in two or more subcellular location sites is an important issue for understanding the biological process in a cell. A recent review summarizes the prediction methods that can deal with proteins with multiple locations (Chou, 2019).

A number of differentially localized isoform pairs were found by MS-based approaches (Christoforou et al., 2016; Geladaki et al., 2019). Such localization change at the isoform level is an interesting issue in terms of targeting signal usage. Protein isoforms seem to be generated by a stress response or in a tissue-specific manner. Thus, a number of localization changes at the isoform level may have been unidentified still. For mitochondrial proteins, we previously applied MitoFates to search for differentially-localized candidates of isoforms and obtained 517 genes, which were 44% of the predicted mitochondrial genes (Fukasawa et al., 2015), suggesting that the major localization changes of mitochondrial protein isoforms are regulated by the changes in their N-terminal targeting signal. Recently, relative protein levels of more than 12,000 genes across 32 normal human tissues were quantified and tissue-specific or tissue-enriched proteins were identified (Jiang et al., 2020). Also, they identified a total of 2,436 tissue-enriched protein isoforms. Those isoforms may be useful for the investigation of tissue-specific localization changes at the isoform level.

Multiple localization proteins and localization changes among isoforms imply potential “moonlighting” activity. Comprehensive analyses of these proteins should boost our further understanding in cell biology.

## Conclusion

A number of computational tools for the analyses of protein subcellular localization are introduced in this review. Although many of the localization sites of a given protein would be able to be predicted through a mere homology transfer nowadays, we would like to emphasize that the subcellular localization prediction problem is not a pedantic one at all. The authors believe that the *in silico* accumulation of various knowledge on protein sorting/targeting processes is important. Prediction methods can be used for assessing how much we understand these processes quantitatively. The future methods should be useful for various purposes, such as for the evaluation of artificial proteins, for understanding why some proteins are localized at multiple positions and for inferring how tissue-specific and/or condition-specific isoforms can change their localization sites. Therefore, in our opinion, the knowledge-based approach would be most important in the future of this field and such knowledge should be integrated into the wider knowledge on the *in vivo* fate of proteins since all of the processes are interrelated with each other (Nakai, 2001).

## Author Contributions

Both the authors listed have made a substantial, direct and intellectual contribution to the work, and approved it for publication.

### Conflict of Interest

The authors declare that the research was conducted in the absence of any commercial or financial relationships that could be construed as a potential conflict of interest.
